# Potential Role of Anti-Complement Agents in the Treatment of COVID-19-Related ARDS

**DOI:** 10.31138/mjr.31.3.304

**Published:** 2020-09-08

**Authors:** Christina Spanopoulou, Dimitrios Kassimos, Dimitrios Cassimos

**Affiliations:** 1 Department of Internal Medicine, General Hospital of Amaliada, Amaliada, Greece; 2 Paediatric Department, Democritus University of Thrace, Alexandroupolis, Greece; 3 Paediatric Clinic, General University Hospital of Alexandroupolis, Alexandroupolis, Greece

**Keywords:** anti-C5a, COVID-19, ARDS, complement system, cytokine storm

To the Editor,

The current COVID-19 pandemic has caused hundreds of thousands of deaths with fatality figures continuing to rise. Lung injury has been the leading cause of mortality with nearly 90% of deaths resulting from ARDS. This condition is a serious immunological complication, characterised by widespread lung inflammation causing severe hypoxaemia, refractory to oxygen treatment. To date, no pharmacological treatment has been approved for the management of ARDS. So far, mechanical ventilation with the application of positive end-expiratory pressure (PEEP) has been the standard of care.^1^

As it has recently been established, hyperinflammation, also described as “cytokine storm”, holds a significant role in the pathogenesis of severe COVID-19. In patients admitted to intensive care units (ICU), higher levels of cytokines have been detected in plasma samples, with the cytokine storm being proportional to disease severity. This observation raises the possibility of using immunosuppressive medications in the treatment of patients with signs of respiratory failure. Given the inflammatory excess implicated in SARS-CoV-2-induced ARDS, numerous clinical trials are investigating the use of cytokine inhibitors targeting specific inflammatory markers such as IL-1β, IL-6, IFNg and TNFa. Of those, tocilizumab, a recombinant humanised monoclonal antibody that acts as an IL-6 receptor antagonist, has been reported to improve the clinical outcome of patients with COVID-19-related lung disease. In addition, JAK-STAT signalling inhibitors, approved for rheumatoid arthritis and myelofibrosis, have been suggested for the prevention of cytokine storm in COVID-19 and other severe viral infections. Lastly, corticosteroids and intravenous immunoglobulin are also being evaluated as candidate therapeutic agents.^2^

Further investigations on the role of the complement in the initiation of the cytokine storm are required. Prior research on highly pathogenic viruses like SARS-CoV has indicated an exaggerated complement activation, and a subsequent cytokine storm, to be implicated in the pathogenesis of lung injury.^3^ Therefore it is fair to hypothesize that blockade of complement signalling could be a promising treatment strategy for the alleviation of SARS-CoV-2-induced ARDS.

The complement system is a key element of innate immunity whose dysregulation is involved in various pathologic conditions including inflammatory, autoimmune, and infectious diseases. Anaphylatoxin C5a is one of the most potent proinflammatory mediators. Its strong chemoattractant properties are involved in the recruitment of neutrophils and macrophages which promotes vascular leakage and protein accumulation in the airways, thus contributing to tissue damage.^4^

Based on earlier reports, bronchoalveolar lavage fluid collected from patients infected with H1N1 virus has been found to include increased levels of C5a. Reversely, C5a-dependent chemotactic activity is significantly decreased in recovered patients.^5^

More recently strong correlations between complement suppression and resolution of lung inflammation have been shown. In a murine model of MERS-CoV infection complement inhibition not only decreased tissue damage but also reduced viral replication in lung tissues.^6^ In another study IFX-1, an anti-complement C5a monoclonal antibody, reduced serum levels of TNF-α, IL-1β, and IL-6 in African Green Monkeys infected with H7N9 virus.^7^

Currently, German biopharmaceutical company InflaRx is conducting an open-label randomized phase II/III clinical trial of IFX-1, after initially obtaining positive preliminary results from patients with severe SARS-CoV-2-induced pneumonia treated with Toll-like receptor agonist named BDB-001.^8^

However, the use of immunosuppression during viral infections faces the challenge of possible disruption in mounting adaptive immunity and impairment of anti-viral response. Furthermore, there is concern that following anti-complement therapy, there is increased risk of bacterial superinfections, given that patients deficient in classical, lectin or alternative pathway components present with predisposition to recurrent respiratory infections and infections caused by encapsulated organisms.^9^

Yet, data from a phase III clinical trial of Anakinra in patients with sepsis showed significant survival benefit without higher incidence of adverse events. This is further supported by randomised studies in critically ill patients treated with anti-TNF agents, which indicate that monoclonal therapy is safe with benefits outweighing risks when administrated to cases with severe disease.^10^ To determine the subgroup which would most benefit from immunosuppression, it is advised for patients to be screened for laboratory findings consistent with hyperinflammation (elevated ferritin and erythrocyte sedimentation rate and decreased platelet counts), similarly seen in those with secondary hemophagocytic lymphohistiocytosis.^11^

In conclusion, the SARS-CoV-2 pandemic poses a serious threat to public health and a major therapeutic challenge for clinicians. In light of the accelerating demand for effective treatment interventions, repurposing of existing medications remains the mainstay of medical research. Dysregulated complement activity is involved in excessive cytokine release, which promotes lung tissue damage. It is therefore evident that anti-complement therapy in patients with ARDS warrants consideration. We believe that combination treatment with antiviral and antibacterial medications will maximise therapy outcomes if promptly applied. Given the rapid progression of pneumonia to acute lung injury, early administration of immunosuppression could have a huge impact on prognosis both by reducing disease severity and mortality, and by shortening ICU stay in ventilated patients.
